# Social Factors Predict Distress Development in Adults With Pre-existing Mental Disorders During the Coronavirus Disease 2019 Pandemic

**DOI:** 10.3389/fpsyg.2022.849650

**Published:** 2022-07-01

**Authors:** Annika C. Konrad, Katharina Förster, Marcel Kurtz, Tanja Endrass, Emanuel Jauk, Philipp Kanske

**Affiliations:** ^1^Clinical Psychology and Behavioral Neuroscience, Institute of Clinical Psychology and Psychotherapy, Faculty of Psychology, Technische Universität Dresden, Dresden, Germany; ^2^Addiction Research, Institute of Clinical Psychology and Psychotherapy, Faculty of Psychology, Technische Universität Dresden, Dresden, Germany; ^3^Department of Medical Psychology and Psychotherapy, Medical University of Graz, Graz, Austria

**Keywords:** COVID-19, psychological distress, pre-existing mental disorders, social resources, empathy, social isolation

## Abstract

Physical distancing measures during the coronavirus pandemic are associated with increased psychological distress, especially in people with mental disorders. We investigated which social risk and resilience factors influence distress over time in people with pre-existing mental disorders. We conducted a longitudinal online survey with weekly follow-ups between April and July 2020 (*n* = 196 individuals with, and *n* = 545 individuals without pre-existing mental disorders at baseline). Our results show that individuals with, but not those without pre-existing mental disorders displayed higher distress levels when social resources and empathic disconnection are low and perceived social isolation is high. The distress development differed between participants with and without pre-existing mental disorders depending on their level of social resources, empathic disconnection, and perceived social isolation. These findings offer specific information for targeted social interventions to prevent an increase in incidence of mental disorders during physical distancing measures.

## Introduction

The coronavirus disease 2019 (COVID-19) necessitates wide-ranging changes in our social lives, such as physical distancing measures. Considering the importance of physical closeness for human well-being, research agrees that distancing has a negative effect on mental health (Murphy M. et al., 2018). Several recent studies have shown an association between physical distancing during the COVID-19 pandemic and an increase in psychological distress, depressive symptoms, and anxiety (Fernández et al., 2020; McGinty et al., 2020; Petzold et al., 2020; Vindegaard and Benros, 2020; for a meta-analysis see Prati and Mancini, 2021). Especially individuals with pre-existing mental disorders seem to be at risk for symptom exacerbation (Fernández et al., 2020; Shi et al., 2020; Robillard et al., 2021; for review, see Vindegaard and Benros, 2020).

However, in a recent meta-analysis mental health before and during the pandemic was compared, but there was no evidence for a general aggravation of mental health symptoms in individuals with pre-existing mental disorders (Robinson et al., 2022). For instance, Pan et al. (2021) found higher overall symptom burden in individuals with vs. without pre-existing mental disorders *during*, but no or only a slight increase in their symptom severity compared to *before* the pandemic. Yet, there is conflicting evidence whether individuals with compared to without pre-existing mental disorders exhibited different symptom trajectories over the course of the pandemic (for current literature see Supplementary Table S1). Whereas some authors reported an association of a history of mental disorders with worse symptom course (Pierce et al., 2021; Yocum et al., 2021), others reported that symptom burden did not change in those with or without a chronic mental disorder (Mergel and Schützwohl, 2021). These results suggest that individuals with a history of mental disorders exhibit *high* variance in their ability to cope, either adaptively or maladaptively, with collective life events in the long run.

Up to now, there is little evidence on specific risk factors that increase the vulnerability for worse symptom trajectories within this high-risk group during the COVID-19 pandemic (Sergeant et al., 2020). A better understanding of modulatory risk and resilience factors is crucial to specifically target prevention strategies during times of collective crises, depending on the individual risk profile. For instance, female gender, individuals with specific disorders as well as with comorbid mental disorders within the group of people with pre-existing mental disorders seemed to be associated with a worse course of symptoms over time (Bartels et al., 2021; Bendau et al., 2021). Next to these risk factors, specific social risk and resilience factors that may modulate symptoms over time within people with and without pre-existing mental disorders and thus could explain variance between study results, have been rarely investigated.

Considering preceding laboratory research, different studies have suggested that (psychological) distress and symptom severity are influenced by a number of social factors including self-perceived social isolation, lack of social resources (Wang et al., 2018), and empathic abilities (Engert et al., 2014). Regarding the latter, being overly involved with others’ emotions (low empathic disconnection) seems to be a risk for increased distress, also on an endocrine level (Engert et al., 2014), and is associated with psychopathology (Hoffmann et al., 2016; Trautmann et al., 2018).

Integrating these research findings in vulnerability-stress models will provide a theoretical framework for probing the modulatory effects of social trait (e.g., social resources and empathic disconnection) and state factors (e.g., subjective social isolation) on psychological distress. The COVID-19 related physical distancing measures, allow testing such models in a naturalistic setting, which can improve our understanding of relevant social factors that affect trajectories of mental well-being, especially in high-risk groups. Moreover, investigating the effects of social risk and resilience factors during the COVID-19 pandemic in individuals with and without pre-existing mental disorders will extend prior research by shedding light into the variation of different symptom trajectories of people at risk.

For this reason, this study asks how social factors, such as social resources, empathic disconnection, and social isolation, influence psychological distress over time in interaction with pre-existing mental disorders. Specifically, we ask if people with pre-existing mental disorders show, first, different psychological distress levels in average, and second, different time patterns of psychological distress depending on their level of social resources, empathic disconnection, and social isolation.

## Materials and Methods

### Recruitment

We conducted a longitudinal online survey with weekly follow-ups from April 3 to July 1, 2020. Participants were recruited online via circular emails, social media, and the mailing list of the university. They were asked to participate in the online study to help investigate psychological stress during the COVID-19 pandemic. At the end of the baseline survey, they were invited to subscribe to a mailing list in order to receive all weekly follow-up surveys. After the end of study, *n* = 10 participants who participated in at least one of the follow-up surveys were randomly drawn in a lottery and received a voucher of 30€.

The authors assert that all procedures contributing to this work comply with the ethical standards of the relevant national and institutional committees on human experimentation and with the Helsinki Declaration of 1975, as revised in 2013. The study was approved by the local ethics committee (SR-EK-302072020). Written informed consent was obtained from all participants.

### Measures

**Figure 1:**
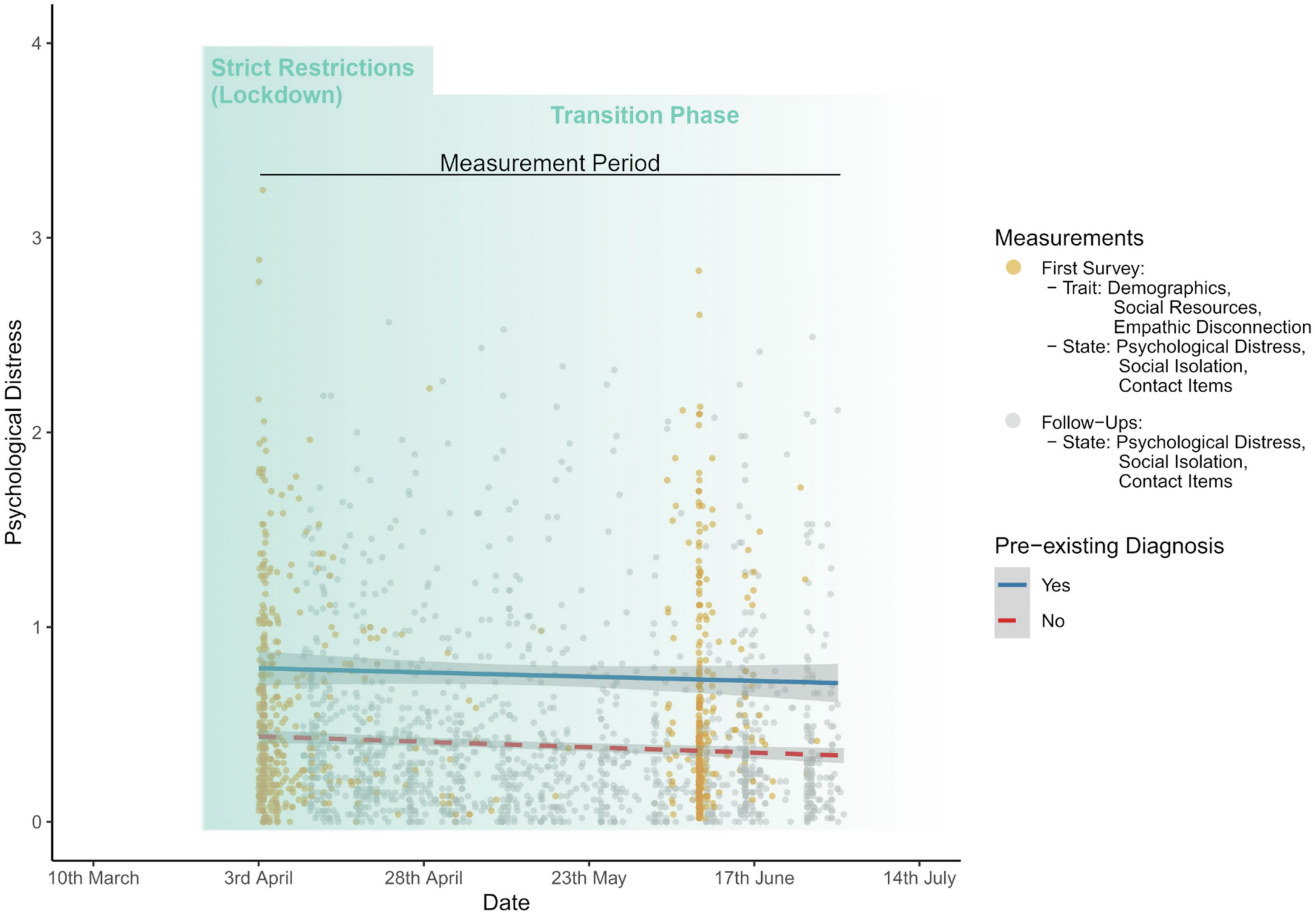
Observations and mean psychological distress levels over time for individuals with and without pre-existing mental disorders during lockdown and transition phase of the coronavirus disease 2019 (COVID-19) pandemic in Germany. In total, *n* = 2,144 observations are displayed grey and yellow dots.

Figure 1 depicts the number of observations and average distress levels of participants over time.

At baseline, we assessed demographics, such as age, gender, and number of household members. All participants were asked to select all categories (*yes* vs. *no*) that applied to their current work situation (e.g., home office, loss of job, and extra hours). We also asked for life-time diagnosis of a mental disorder, assessed by a clinician (*yes* vs. *no*), type of diagnosis, stationary treatment due to a mental disorder (lifetime), current psychological treatment (*yes* vs. *no*), and for specification of the current treatment (psychotherapy, counselling, psychiatric treatment, and other treatments, such as digital counselling or no psychotherapy).

Two trait social factors were only acquired at baseline: *empathic disconnection* was assessed with the emotional disconnection subscale of Brief Empathy Scale (BES; Jolliffe and Farrington, 2006; Carré et al., 2013, Cronbach’s alpha at baseline *α* = 0.72). The subscale has six items (e.g., “Seeing a person who has been angered has no effect on my feelings.”) that are rated by participants on a five-point response scale ranging from *strongly disagree* (1) to *strongly agree* (5). We used a mean score with higher values indicating more empathic disconnection. As a second social trait factor, we assessed *social resources* at baseline with the social resources subscale of Resilience Scale for Adults (RSA; Friborg et al., 2003, Cronbach’s alpha at baseline *ɑ* = 0.78). The subscale has seven items. Participants rate the displayed statements on a seven-point response scale, whereas each item has two individual verbal anchors (e.g., “I can discuss personal topics with …” with 1 = *no one* to 7 = *with friends or family members*). Higher levels on the subscale indicate a higher degree of social resources.

The third social factor, we were interested in, was *perceived social isolation* as a state that was assessed at baseline and each follow-up with the social isolation subscale of Trier Inventory for Chronic Stress (TICS, adapted to assess the last 7 days; Schulz et al., 2004, Cronbach’s alpha at baseline *ɑ* = 0.84). The subscale consists of six items (e.g., “Times where I often was alone”) that are displayed on a five-point response scale, ranging from *never* (0) to *a lot* (4). Here, we used a mean score with higher values indicating higher levels of perceived social isolation.

As our primary outcome variable, we assessed at baseline and at each follow-up *psychological distress* with the Global Severity Index (GSI) of the Brief Symptom Inventory (BSI, adapted to assess the last 7 days; Derogatis, 1993, Cronbach’s alpha at baseline *ɑ* = 0.96). The scale consists of 53 items that cover multiple psychological and body-related symptoms (e.g., “Feeling no interest in things”). Participants were asked to rate each item on a five-point response scale, ranging from *not at all* (0) to *extremely* (4). The GSI was calculated by using a mean score of all items, whereas higher GSI levels mean higher overall symptom burden.

Moreover, as control variables, we asked participants at baseline and each follow-up for digital contacts with family and friends (averaged minutes per day during the last week) and the amount of real-life contacts with family and friends (number of contact-days per week with >15 min, within 2 m of distance) during the last 7 days with self-designed questions assessing objective isolation. Scores for family and friends were averaged.

### Statistical Analysis

All analyses were conducted with R Core Team (2021). We aimed to investigate interaction effects of time, pre-existing diagnoses of mental disorders, and different trait and state social factors (*trait:* social resources, empathic disconnection; *state:* subjective social isolation) on psychological distress by applying mixed-effects multi-level models.

Due to minimum and maximum problems regarding the variables age, real-life contact per week and digital contact per day, we excluded *n* = 7 observations from a total of *n* = 2,144 observations when analyzing the social resource model (model 1) and the empathic disconnection model (model 2), and *n* = 18 observations when analyzing the social isolation model (model 3). Shapiro–Wilk test statistics of normality of numeric variables are displayed in Supplementary Table S2. Group differences (individuals with vs. without pre-existing mental disorders) were computed by using Mann–Whitney *U* tests for all numeric variables and Pearson’s Chi-squared tests for categorical data. Effect sizes (*r* for Mann–Whitney *U* tests, *Φ* for 2-by-2 and Cramer’s *V* for 2-by-3 contingency tables) are depicted in Table 1. Since the expected frequency for the category “other/non-binary” gender was very small (<5), Yates’ continuity correction was applied.

**Table 1:** Descriptive statistics and demographics at first survey (Baseline).

	Participants with pre-existing mental disorder (*n* = 196)	Participants without pre-existing mental disorder (*n* = 545)	Statistics	*p*	Effect size
	Median [IQR]^a^	Median [IQR]^a^	*Z*		*r*
Age (in years)	35.0 [27.0, 45.0]	33.0 [26.0, 45.0]	0.68	0.498	0.02
Psychological distress (GSI)	0.7 [0.3, 1.3]	0.3 [0.2, 0.6]	8.43	<0.001	0.31
Social isolation (TICS)	2.0 [1.3, 2.7]	1.5 [0.8, 2.3]	4.41	<0.001	0.16
Social resources (RSA)	5.7 [5.0, 6.4]	6.1 [5.4, 6.6]	−3.54	<0.001	0.13
Empathic disconnection (BES)	2.0 [1.7, 2.5]	2.2 [1.8, 2.5]	−2.34	0.019	0.09
Number of household members	2.0 [2.0, 4.0]	2.0 [2.0, 3.0]	0.12	0.907	0.00
Real-life contact with family and friends (days per week)	1.5 [0.5, 3.5]	1.5 [0.0, 3.5]	−0.30	0.763	0.01
Digital contact with family and friends (minutes per day)	51.2 [22.5, 105.0]	40.0 [20.0, 90.0]	1.37	0.172	0.05
	**No. (%)**	**No. (%)**	***Χ***^**2**^ **(df)**		**Cramer’s** ***V***
Gender^b^			2.71 (2)	0.257	0.06
Female	149 (76.0)	380 (69.7)			
Male	45 (23.0)	160 (29.4)			
Other	2 (1.0)	5 (0.9)			
Current treatment setting			105.39 (2)	<0.001	0.38
Current psychotherapy, counselling, or psychiatric treatment	33 (16.8)	10 (1.8)			
No Psychotherapy	121 (61.7)	502 (92.1)			
Other (e.g., digital meetings)	42 (21.4)	33 (6.1)			
					***Φ***
Home office (yes)	116 (59.2)	341 (62.6)	0.70 (1)	0.403	0.03
Loss of job (yes)	9 (4.6)	18 (3.3)	0.68 (1)	0.409	0.03
Extra hours (yes)	10 (5.1)	23 (4.2)	0.26 (1)	0.608	0.02
Stationary treatment (yes)	58 (29.6)	6 (1.10)	148.29 (1)	<0.001	0.45
Diagnosis category^c^					
Depression	50 (26.2)				
Anxiety disorder	17 (8.9)				
Eating disorder	9 (4.7)				
PTSD	12 (6.3)				
AD(H)D	6 (3.1)				
Adjustment disorder	19 (10.0)				
Other mental disorder	11 (5.8)				
Comorbid disorders	52 (27.2)				
Answer could not be assigned to any category	15 (7.9)				

GSI, Global Severity Index; TICS, Trier Inventory for Chronic Stress; RSA, Resilience Scale for Adults; BES, Brief Empathy Scale; Effect sizes indicate a small effect with *r*, Phi (Φ) and Cramer’s *V* < 0.3 and a moderate effect with 0.3 ≤ *r*, Phi (Φ) and Cramer’s *V* < 0.5 (Cohen, 1988).

a Non-normal distributed variables are displayed with median (IQR = interquartile range) and group differences were tested with Mann–Whitney U tests.

b In this case, Pearson’s Chi-squared test was adjusted with Yates’ continuity correction.

c *n* = 10 individuals without pre-existing mental disorder reported mental health problems or assumptions about a diagnosis they might have (e.g., hypochondria) without ever having been officially diagnosed.

We first calculated non-robust multilevel models with “lme4” R package (Bates et al., 2015). We started with simple random intercept models and then added stepwise covariates, fixed effects predictors, and finally interaction terms. Thus, in each of the three final models, we predicted psychological distress by a three-way-interaction, respectively [Social Factor × Diagnosis of a pre-existing mental disorder (yes vs. no) × Time (relative to study start)], adjusted for age and gender. The social isolation model was additionally controlled for objective isolation measures (household-size, digital and real-life contact with family and friends).

Checking the final models for assumptions revealed issues with heteroscedasticity and the assumption of normally distributed errors. To address this, we refitted the full random intercept models with “robustlmm” R package (Koller, 2016) The package uses a smoothed Huber function to receive more robust variance components and random effects (e.g., by down-weighing outliers). For model fits, we used default options (computation method: DAStau, smoothed Huber function with tuning parameters: *k* = 1.345 and *s* = 10). The provided *p* values of the regression estimates are based on the Wald approximation method. Additionally, we computed standardized regression estimates of the robust models using the *standardize()* function of the “datawizard” R package (Makowski et al., 2021). All predictors were scaled by two times their standard deviation (variablewise standardization). Through this method, estimates of numeric and binary predictors are better comparable (Gelman, 2008). Since our models included interaction terms, Variance Inflated Factors (VIFs) as indication for multicollinearity were likely to be inflated. However, multicollinearity for the standardized models were in a low to moderate range (all VIFs < 5.2; James et al., 2021).

To receive a better understanding of resulting interactions, we refitted the models for participants with and without pre-existing mental disorders, separately, and used pairwise comparisons with Bonferroni adjusted *p* values to analyze distress trends and averaged distress levels over time, each at fixed social factor levels (mean, +/−*SD*). For all trend analyses, non-standardized variables were used, because the unstandardized time variable was easier to interpret.

Finally, we conducted sensitivity analyses and refitted all models while excluding observations of individuals without pre-existing mental disorders that reported, first, stationary treatment in their lifetime because of mental health problems (*n* = 7 observations), second current psychiatric, psychotherapeutic treatment or counselling (*n* = 28 observations), or third, a mental disorder, without ever being officially diagnosed (*n* = 44 observations). Model results of the refitted models did not differ from original models, which is why we report the original models.

## Results

### Participant Characteristics

Table 1 shows descriptive statistics and between-groups comparisons (individuals with vs. without pre-existing mental disorders) of demographic, clinical, and social variables. In total, we recruited *n* = 741 participants at baseline (55.7–1.2% participation from 1st to 10th follow-up, average participation in follow-ups = 3.2 follow-ups). From these participants, *n* = 196 individuals reported a pre-existing (remitted or current) diagnosis of one or more mental disorders with depression as most frequently reported disorder (*n* = 50; see Table 1).

The groups did not differ regarding relevant demographic variables, such as age, gender distribution, or work-related variables (all *p*s > 0.05). Participants with pre-existing mental disorder exhibited significant higher levels in all relevant clinical and social variables at baseline (all *p*s < 0.05 with small to moderate effect sizes).

### Main Results of Multilevel Models

**Figure 2:**
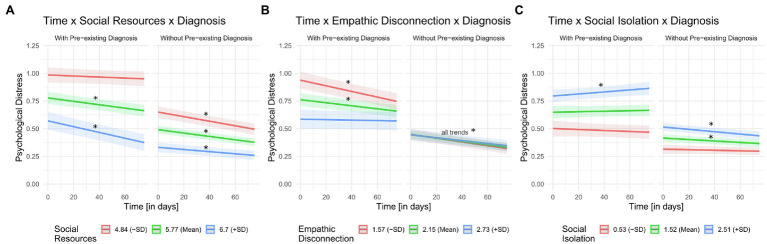
Three-way interactions between time, current, or preceding diagnosis of a mental disorder and **(A)** social resources, **(B)** empathic disconnection, and **(C)** social isolation. For visual purposes, we split the continuous moderator variables **(A)** social resources, **(B)** empathic disconnection, and **(C)** social isolation into mean and mean ±one SD to plot interaction terms. Also for visual purposes, time variable and social moderator variables were not standardized. Standardization vs. not standardization of variables did not have an effect on the displayed results. * means significant time trend (*p* < 0.01). Shaded areas represent 95% confidence bands.

All three models showed significant three-way interactions (see Figure 2), while controlling for age, gender (model 1 and 2), and additionally for objective isolation measures (model 3; for full model statistics of all three models see Supplementary Table S3). Thus, individuals with and without pre-existing mental disorders differ in their development of distress over time, depending on the level of social isolation [model 1: *ß* = 0.38, 95% CI [0.23, 0.53], *t*(2,126) = 4.93, *p* < 0.001], empathic disconnection [model 2: *ß* = −0.23, 95% CI [−0.40, −0.06], *t*(2,126) = −2.64, *p* = 0.008], and self-perceived social isolation [model 3: *ß* = −0.26, 95% CI [−0.41, −0.11], *t*(2,112) = −3.31, *p* = 0.001]. Analyzing the models for individuals with and without history of mental disorder separately, confirmed the results of different distress trajectories depending on the extent of social factors (see Supplementary Tables S4–S6).

#### Model 1: Social Resources

Posttests revealed, first, that individuals with pre-existing mental disorders exhibited higher distress when having *low* social resources (compared to *average* and *higher* social resources *within-group* and compared to all social resource levels *between-group*, all *p*_adj._ < 0.001, see Figure 2A and Supplementary Table S8). Second, distress trajectories over time differed between groups: individuals with pre-existing mental disorders only showed a significant decrease in distress over time when also reporting *average* or *high* social resources, but not when having *low* social resources. Regarding the latter, distress levels were persistently high over time. For individuals without pre-existing mental disorders a distress decrease was found for all levels of social resources (Supplementary Table S7).

#### Model 2: Empathic Disconnection

Individuals with pre-existing mental disorders showed higher average distress when having *low* empathic disconnection (compared to *average* and *higher* empathic disconnection *within-group* and compared to all empathic disconnection levels *between-group*, all *p*_adj_. < 0.001, see Figure 2B and Supplementary Table S10). Despite showing initially higher distress levels, individuals with pre-existing mental disorders and *low* or *average* empathic disconnection showed a decrease in distress over time. Individuals without pre-existing mental disorders did not differ in their distress levels depending on empathic disconnection abilities (all *p*_adj._ = 1.00), and a distress decrease was found for all levels of empathic disconnection (see Supplementary Table S9). This result was reflected in the analysis separated per group. Here, the main effect of time on psychological distress was significant (*p* < 0.001), but neither the main effect of empathic disconnection (*p* = 0.521) nor the interaction effect of time and empathic disconnection (*p* = 0.135, Supplementary Table S5).

#### Model 3: Social Isolation

Regarding the social isolation model, participants with pre-existing mental disorders and *high* social isolation exhibited higher psychological distress (compared to *average* and *low* self-perceived isolation within-group and compared to all social isolation levels between-group, all *p*_adj._ < 0.001, see Figure 2C and Supplementary Table S12). We did not find a change of distress levels in individuals with pre-existing mental disorders for *low* and *average* levels of social isolation, but a small increase in distress for *high* social isolation (Supplementary Table S11). In contrast, participants without pre-existing mental disorders did show a decrease in distress over time when reporting *high* and *average* social isolation.

## Discussion

The current study aimed to probe modulatory effects of social factors, pre-existing mental disorders, and time on psychological distress during the COVID-19 pandemic. By conducting a prospective longitudinal online survey, our study could investigate time patterns and interacting effects of psychological distress in a convenience sample of individuals at risk. Our results indicate that all participants show higher distress levels when they are *low* in social resources and *high* in social isolation (irrespective of objective isolation measures). But only participants with, not those without, pre-existing mental disorders displayed high distress levels when reporting *low* empathic disconnection.

We further investigated the interplay of different risk and resilience factors with pre-existing mental disorders over time. By doing so we supplemented other studies that also explored potential risk factors for unfavorable symptom trajectories (Bartels et al., 2021) and focused especially on social factors. Here, we found that individuals with and without pre-existing mental disorders exhibit a different development of psychological distress depending on their expression in social risk and resilience factors. In line with other studies (Bartels et al., 2021; Bendau et al., 2021), changes of psychological distress over time were rather small with a decreasing trend from March to July 2020.

### Psychological Distress

Interestingly, psychological distress of individuals without a history of mental disorders at baseline was below the clinical cut-off of GSI ≥ 0.62 and, thus, comparable to distress levels of a healthy norm sample of adults (Franke, 2000). Individuals with pre-existing mental disorders exhibited baseline distress levels that were higher than the clinical cut-off (Franke, 2000). Compared to a sample of Moroccan adults during quarantine (Sfendla and Hadrya, 2020), who reported about three-times larger levels of psychological distress (measured with the GSI) the levels in our sample were still relatively low. This difference might be because full quarantine and lockdown measures are not the same and most of our participants were not in quarantine throughout measurement. Nevertheless, our results suggest that average distress in people without pre-existing mental disorder in Germany was not largely elevated during the first lockdown, while it was elevated for people with pre-existing mental disorders. However, we cannot rule out that, similar to other longitudinal studies, symptom burden within individuals with pre-existing mental disorders did not increase but remained stable on a high level compared to before the pandemic (Pan et al., 2021; Racine et al., 2021; for meta-analysis see Robinson et al., 2022).

### Social Resources

Regarding the role of social resources, we found, first, that both groups (individuals with and without pre-existing mental disorders) displayed lower distress when they also reported *average* or *high* social resources compared to those who reported low social resources.

Second, only the combination of having *low* social resources and pre-existing mental disorders was associated with persistently high psychological distress throughout the measurement period. In contrast, psychological distress decreased over time for all other groups (for all levels of social resources in individuals without pre-existing mental disorders and for high and average levels of social resources in individuals with pre-existing mental disorders).

These findings demonstrate that higher social resources do not only protect individuals from initial high distress during a strict lockdown but also lead to an attenuation of distress over time. Low social resources seem to be a risk factor for persistently high psychological distress in individuals with, but not without, pre-existing mental disorders.

This is in line with other findings, demonstrating that poorer social support in individuals with depression predicts consistently negative mental health outcomes, such as greater symptom severity (for review, see Wang et al., 2018). Moreover, one previous study demonstrated that patients with depression and suicide risk reported lower levels of subjective social support compared to patients with no risk for suicide, even though the groups did not differ regarding objective (practical) support (Lin et al., 2020). Since depression and other mental disorders are also associated with a negative interpretation bias such as high sensitivity to social rejection (Kupferberg et al., 2016; Gao et al., 2017), individuals with (pre-existing) mental disorders may have problems in perceiving their given social interactions as helpful or caring.

Taken together, our findings have major implications for clinical practice: Especially those people with pre-existing mental disorders and *low* self-perceived social resources should be targeted in prevention and intervention strategies, by strengthening the subjective perception of their given social support system.

### Empathic Disconnection

We investigated empathic disconnection as a second potential social factor that may influence distress in people with pre-existing mental disorders over time. Here we found, first, that *average* and *lower* levels of empathic disconnection and having pre-existing mental disorders yields initially higher distress levels during the strict lockdown. In other words, being empathically overinvolved increases the risk for being particularly affected at the beginning of strict physical distancing measures.

However, regarding time trends, we observed that individuals with pre-existing mental disorders and *low* empathic disconnection showed a decrease in psychological distress, indicating an adaption over time. Only those with pre-existing mental disorders and *high* empathic disconnection showed no decrease in psychological distress, but persistently low distress levels.

Our findings are broadly in line with two research lines in the field of empathy research. Some authors have discussed *empathic disconnection* on a conceptual level as a coping strategy enabling an emotional distance from others’ suffering and hence reducing own empathic distress (Regehr et al., 2002). However, our results indicate that only in individuals with (but not without) pre-existing mental disorders, did empathic disconnection serve as coping strategy, yielding initially, and constantly *low* distress levels during a collective crisis.

A second research line has been focusing more on the reverse construct, specifically *emotional contagion*, describing it as a double-edged process. Empathic distress can derive from the contagion of negative emotions, but conversely, someone can also become “infected” with positive emotions and experience positive affect on their own (Murphy B. et al., 2018). Here, we demonstrated that individuals with high emotional contagion (*low* empathic disconnection) and pre-existing mental disorders are initially highly distressed, which is in line with other findings (Wheaton et al., 2020) and with the high emotional insecurity conveyed by the situation at the beginning of the pandemic.

Additionally, our observation that individuals with, but not without, pre-existing mental disorders show different distress levels depending on their ability to empathically disconnect, also matches previous cross-sectional findings (Derntl et al., 2012; Kupferberg et al., 2016). These studies demonstrated an association of mental disorders (e.g., depressive and bipolar disorders) with higher trait empathic (personal) distress. We extend those results with insights into the time course of distress, since we observed an adaptation and therefore an attenuation of distress over time, despite initial high (empathic) distress.

Summarizing our findings, *low* empathic disconnection (thus, *high* empathic contagion) only seems to be a social risk factor for high distress in individuals with pre-existing mental disorders in the short-, but not in the long-term.

### Self-Perceived Social Isolation

Perceived isolation was identified in multiple studies as a general risk factor for poorer mental health during the pandemic (Hofer et al., 2022; for review, see Wirkner et al., 2021), but our findings add new insights into the differential effects of perceived social isolation on the time course of psychological distress. Looking closer at our results, those participants with pre-existing mental disorders and *higher* self-reported social isolation also exhibited higher distress. Moreover, we did find a small increase of distress in individuals with pre-existing mental disorders with high levels of perceived social isolation. In contrast, while individuals without a history of mental disorders also show initially higher distress levels when reporting *high* social isolation, they do recover over time.

The absence of an interaction effect in the separate analysis confirms the vast, persistent negative effect of subjective isolation experiences on mental health in already vulnerable individuals (Jeong et al., 2016). In line with this, a large, cross-cultural study did show that individuals with compared to without previous mental disorder reported higher levels of loneliness during the first months of the pandemic (Varga et al., 2021). Interestingly, there are contrasting findings of a small longitudinal study, showing that individuals with an acute or chronic mental disorder did not report changes in their social participation and inclusion between March and July 2020 (Mergel and Schützwohl, 2021). These two studies cannot be compared directly, since they used different measures, but still emphasize the variety of psychological reactions to the COVID-19 pandemic among people at risk. Therefore, interventions should consider self-perceived social isolation in general as a risk for distress in people with mental health issues and acknowledge individual variation. Additionally, our effects were controlled for objective social isolation measures, highlighting the importance of discriminating between these two measures of isolation.

### Strengths and Limitations

Limitations of our study include the drop-out over time and the self-report assessment of mental disorders. Due to the fast-changing situation at the beginning of the pandemic, we were not able to conduct structured interviews. Also, the data did not allow to discriminate between individuals that currently meet the diagnostic criteria for a mental disorder and those that are in remission. Our sample was a convenience sample recruited at the beginning of the lockdown measures, so we were not able to compare the results to data prior to the lockdown. We also did not collect data on racial or ethnic identification, and cultural background. The time effects we found were small, presumably due to small variance in our outcome variable, and thus, should be interpreted carefully.

Nevertheless, as a prospective longitudinal study, our findings provide essential insights into the etiological relevance of social risk and resilience markers, especially in the development of psychological distress over time, extending previous experimental research to naturalistic settings (O’Connor et al., 2020; Shi et al., 2020). Additionally, we extended current cross-sectional findings by highlighting modulatory effects of psychological distress during and after the strict lockdown phase in Germany in individuals with pre-existing mental disorders. In line with other longitudinal studies (Bartels et al., 2021; Yocum et al., 2021), we observed a decrease in symptom burden in most individuals at risk, but also expanded previous findings by highlighting that the extent of these time trends was depended on specific social factors. We therefore demonstrated that in these vulnerable individuals protective and risk factors can increase or decrease the risk for high psychological distress.

## Conclusion

Given the prolonged duration of the pandemic with ongoing distancing measures, our results permit the identification of specific risk and resilience factors in high-risk groups, informing targeted prevention and intervention. More specifically, clinical practitioners could especially try to help individuals improving their focus on the quality of given relationships in order to reduce self-perceived feelings of isolation. Also, since empathic contagion seemed to be associated with high distress levels in the first days of lockdown in individuals with pre-existing mental disorders, strategies to attenuate negative emotions, e.g., functional reappraisal or compassionate thinking strategies might be helpful (Klimecki and Singer, 2012; Arimitsu and Hofmann, 2017). In summary, future preventive measures should strengthen social resources and cohesion to reduce self-perceived isolation and distress, especially in people with pre-existing mental disorders. These factors and previously identified risk factors, such as female gender or comorbidities (Bartels et al., 2021) seem to prevent a recovery in already vulnerable individuals. Targeting risk factors in online support services, psychotherapy, or counselling could prevent an increase in incidence and relapse of mental disorders in consequence of physical distancing.

## Data Availability Statement

The datasets presented in this study can be found in online repositories. The names of the repository/repositories and accession number(s) can be found at: https://osf.io/8edp7.

## Ethics Statement

The studies involving human participants were reviewed and approved by Ethics Committee TU Dresden. The patients/participants provided their written informed consent to participate in this study.

## Author Contributions

AK, KF, MK, EJ, and PK equally contributed to the study concept, design as well as data acquisition. AK analyzed the data and drafted the manuscript. AK, KF, MK, TE, EJ, and PK provided critical revisions. All authors contributed to the article and approved the submitted version.

## Funding

PK was supported by the German Research Foundation (KA 4412/2-1, KA 4412/4-1, KA 4412/5-1, and CRC940/C07). EJ was supported by the Austrian Science Fund (FWF): J 4344 and the German Research Foundation (CRC940/C07). AK received funding from the Saxon Scholarship Program. The funding bodies had no role in the design, writing or choice to submit this manuscript.

## Conflict of Interest

The authors declare that the research was conducted in the absence of any commercial or financial relationships that could be construed as a potential conflict of interest.

## Publisher’s Note

All claims expressed in this article are solely those of the authors and do not necessarily represent those of their affiliated organizations, or those of the publisher, the editors and the reviewers. Any product that may be evaluated in this article, or claim that may be made by its manufacturer, is not guaranteed or endorsed by the publisher.
