# Supplementary material for: Social Factors Predict Distress Development in Adults With Pre-existing Mental Disorders During the Coronavirus Disease 2019 Pandemic
Source: Front Psychol. 2022 Jul 1;13:849650. doi: 10.3389/fpsyg.2022.849650 (PMC9286674; doi:10.3389/fpsyg.2022.849650)
Supplement: Supplementary file 1 [file Data_Sheet_1.docx]

*Supplementary Material*

# Literature Overview

| Table S1  *COVID-19 Studies Including Longitudinal Mental-health Data from Individuals With Pre-existing Mental Disorders* | | |
| --- | --- | --- |
| Authors | Study type | Design |
| Robinson et al., 2021 | Meta-analysis and review | - Including studies with data before and during the pandemic in 2020 |
| Racine et al., 2021 | Original Paper | - Multiple waves with pre-pandemic data - T1: May 20–July 15, 2020 |
| Pierce et al., 2021 | Original Paper | - Multiple Waves with pre-pandemic data - Five waves during the pandemic (April 24–October 1, 2020) |
| Pan et al., 2021 | Original Paper | - Multiple Waves with pre-pandemic data - T1: April 1–May 13, 2020 |
| Mergel & Schützwohl, 2021 | Original Paper | - Pre-pandemic data - T1: March 23–April 20, 2020 (retrospectively) - T2: June 22–July 19, 2020 (retrospectively) |
| Bartels et al., 2021 | Original Paper | - T1: April/May, 2020 - T2: November/December, 2020 |
| Bendau et al. 2021 | Original Paper | - T1: March 27–April 6, 2020 - T2: April 24–May 4, 2020 - T3: May 15–May 25, 2020 - T4: June 5–June 15, 2020 |
| Hofer et al., 2022 | Original Paper | - T1: June 26–September 13, 2020 and September 7–November 22, 2020 - T2: November 30, 2020– January 24, 2021 and February 8–April 4, 2021 |
| Yocum et al., 2021 | Original Paper | - Pre-pandemic data - T1: April 30, 2020 - T2: May 14, 2020 - T3: May 28, 2020 |
| *Note.* Studies presented here were not systematically reviewed. There is no claim to completeness. | | |

# Descriptive Analysis

| Table S2  *Shapiro-Wilk Normality Tests per Group* | | | | |
| --- | --- | --- | --- | --- |
| Variable | **With pre-existing diagnosis** | | **Without pre-existing diagnosis** | |
|  | *Statistic* | *p* | *Statistic* | *p* |
| Age | 0.947 | **<0.001** | 0.916 | **<0.001** |
| Psychological distress (GSI) | 0.935 | **<0.001** | 0.809 | **<0.001** |
| Social isolation (TICS) | 0.978 | **0.004** | 0.976 | **<0.001** |
| Social resources (RSA) | 0.954 | **<0.001** | 0.909 | **<0.001** |
| Empathic disconnection (BES) | 0.979 | **0.005** | 0.976 | **<0.001** |
| Number of household members | 0.178 | **<0.001** | 0.774 | **<0.001** |
| Real-life contacts | 0.895 | **<0.001** | 0.888 | **<0.001** |
| Digital contacts | 0.687 | **<0.001** | 0.640 | **<0.001** |
| *Note.* GSI = General Severity Index of Brief Symptom Inventory; TICS = Trier Inventory for Chronic Stress; RSA = Resilience Scale for Adults; BES = Brief Empathy Scale. | | | | |

# Robust Models

| Table S3  *Model Comparison* | | | | | | | | | | | | | | |
| --- | --- | --- | --- | --- | --- | --- | --- | --- | --- | --- | --- | --- | --- | --- |
|  | **Model 1** | | | | **Model 2** | | | | | **Model 3** | | | | |
| Predictors | ß | 95% CI | *t* | *p* | ß | 95% CI | *t* | *p* | ß | | 95% CI | *t* | *p* |  |
| (Intercept) | 0.49 | 0.38 – 0.59 | 9.38 | **<0.001** | 0.47 | 0.36 – 0.57 | 8.40 | **<0.001** | 0.37 | | 0.27 – 0.47 | 7.16 | **<0.001** |  |
| Time | -0.18 | -0.25 – -0.11 | -4.98 | **<0.001** | -0.16 | -0.23 – -0.09 | -4.48 | **<0.001** | 0.03 | | -0.05 – 0.10 | 0.75 | 0.456 |  |
| Pre-existing diagnosis [no] | -0.60 | -0.71 – -0.48 | -10.19 | **<0.001** | -0.67 | -0.79 – -0.55 | -10.75 | **<0.001** | -0.56 | | -0.67 – -0.45 | -9.78 | **<0.001** |  |
| Social resources | -1.04 | -1.22 – -0.85 | -10.83 | **<0.001** |  |  |  |  |  | |  |  |  |  |
| Age | -0.18 | -0.28 – -0.07 | -3.24 | **0.001** | -0.18 | -0.30 – -0.07 | -3.14 | **0.002** | -0.16 | | -0.27 – -0.06 | -3.07 | **0.002** |  |
| Gender [male] | -0.22 | -0.33 – -0.10 | -3.78 | **<0.001** | -0.11 | -0.23 – 0.01 | -1.75 | 0.080 | -0.12 | | -0.22 – -0.01 | -2.07 | **0.038** |  |
| Gender [other] | 0.92 | 0.41 – 1.44 | 3.51 | **<0.001** | 1.20 | 0.65 – 1.75 | 4.28 | **<0.001** | 1.09 | | 0.58 – 1.59 | 4.25 | **<0.001** |  |
| Time x pre-existing diagnosis [no] | 0.00 | -0.08 – 0.08 | 0.07 | 0.945 | -0.01 | -0.10 – 0.07 | -0.36 | 0.718 | -0.11 | | -0.19 – -0.02 | -2.48 | **0.013** |  |
| Time x social resources | -0.25 | -0.38 – -0.12 | -3.76 | **<0.001** |  |  |  |  |  | |  |  |  |  |
| Pre-existing diagnosis [no] x social resources | 0.47 | 0.24 – 0.69 | 4.08 | **<0.001** |  |  |  |  |  | |  |  |  |  |
| (Time x pre-existing diagnosis [no]) x social resources | 0.38 | 0.23 – 0.53 | 4.93 | **<0.001** |  |  |  |  |  | |  |  |  |  |
| Empathic disconnection |  |  |  |  | -0.54 | -0.75 – -0.32 | -4.86 | **<0.001** |  | |  |  |  |  |
| Time x empathic disconnection |  |  |  |  | 0.27 | 0.12 – 0.43 | 3.56 | **<0.001** |  | |  |  |  |  |
| Pre-existing diagnosis [no] x empathic disconnection |  |  |  |  | 0.56 | 0.31 – 0.81 | 4.42 | **<0.001** |  | |  |  |  |  |
| (Time x pre-existing diagnosis [no]) x empathic disconnection |  |  |  |  | -0.23 | -0.40 – -0.06 | -2.64 | **0.008** |  | |  |  |  |  |
| Social isolation |  |  |  |  |  |  |  |  | 0.73 | | 0.63 – 0.82 | 14.58 | **<0.001** |  |
| Household members |  |  |  |  |  |  |  |  | -0.00 | | -0.08 – 0.08 | -0.04 | 0.970 |  |

| Table S3 (continued)  *Model Comparison* | | | | | | | | | | | | | |  |
| --- | --- | --- | --- | --- | --- | --- | --- | --- | --- | --- | --- | --- | --- | --- |
|  | **Model 1** | | | | **Model 2** | | | | **Model 3** | | | | |  |
| Predictors | ß | 95% CI | *t* | *p* | ß | 95% CI | *t* | *p* | ß | | 95% CI | *t* | *p* |  |
| Digital contact |  |  |  |  |  |  |  |  | 0.01 | | -0.03 – 0.04 | 0.33 | 0.744 |  |
| Real-life contact |  |  |  |  |  |  |  |  | -0.01 | | -0.05 – 0.03 | -0.49 | 0.627 |  |
| Time x social isolation |  |  |  |  |  |  |  |  | 0.16 | | 0.03 – 0.30 | 2.38 | **0.017** |  |
| Pre-existing diagnosis [no] x social isolation |  |  |  |  |  |  |  |  | -0.38 | | -0.49 – -0.26 | -6.58 | **<0.001** |  |
| (Time x pre-existing diagnosis [no]) x social isolation |  |  |  |  |  |  |  |  | -0.26 | | -0.41 – -0.11 | -3.31 | **0.001** |  |
| Random effects | | | | | | | | | | | | | | |
| σ^2^ | 0.06 | | | | 0.06 | | | | | 0.06 | | | | |
| τ_00_ | 0.41 _id_ | | | | 0.48 _id_ | | | | | 0.39 _id_ | | | | |
| ICC | 0.87 | | | | 0.88 | | | | | 0.87 | | | | |
| N | 739 _id_ | | | | 739 _id_ | | | | | 737 _id_ | | | | |
| Observations | 2137 | | | | 2137 | | | | | 2126 | | | | |
| Marginal R^2^ /  conditional R^2^ | 0.367 / 0.917 | | | | 0.227 / 0.910 | | | | | 0.276 / 0.903 | | | | |
| *Note.* ß = standardized regression estimates. | | | | | | | | | | | | | | |

# Additional Analyses

## Separate Model Analysis

| Table S4  *Analysis of Social Resource Model Separated Per Group* | | | | | | |
| --- | --- | --- | --- | --- | --- | --- |
|  | **With pre-existing diagnosis** | | | **Without pre-existing diagnosis** | | |
| Predictors | ß | 95% CI | *p* | ß | 95% CI | *p* |
| (Intercept) | 0.06 | -0.08 – 0.19 | 0.424 | 0.04 | -0.03 – 0.11 | 0.222 |
| Time | -0.11 | -0.20 – -0.03 | **0.009** | -0.21 | -0.25 – -0.17 | **<0.001** |
| Social resources | -0.86 | -1.10 – -0.61 | **<0.001** | -0.65 | -0.77 – -0.53 | **<0.001** |
| Age | -0.05 | -0.31 – 0.20 | 0.689 | -0.22 | -0.34 – -0.10 | **<0.001** |
| Gender [male] | -0.15 | -0.44 – 0.13 | 0.299 | -0.28 | -0.40 – -0.15 | **<0.001** |
| Gender [other] | 0.59 | -0.57 – 1.76 | 0.317 | 1.20 | 0.59 – 1.81 | **<0.001** |
| Time x social resources | -0.22 | -0.39 – -0.05 | **0.011** | 0.15 | 0.07 – 0.23 | **<0.001** |
| Random effects | | | | | | |
| σ^2^ | 0.09 | | | 0.07 | | |
| τ_00_ | 0.62 _id_ | | | 0.40 _id_ | | |
| ICC | 0.88 | | | 0.85 | | |
| N | 194 _id_ | | | 545 _id_ | | |
| Observations | 517 | | | 1620 | | |
| Marginal R^2^ / conditional R^2^ | 0.228 / 0.904 | | | 0.241 / 0.887 | | |
| *Note.* ß = standardized regression estimates. | | | | | | |

| Table S5  *Analysis of Empathic Disconnection Model Separated Per Group* | | | | | | | |
| --- | --- | --- | --- | --- | --- | --- | --- |
|  | **With pre-existing diagnosis** | | | **Without pre-existing diagnosis** | | | |
| Predictors | ß | 95% CI | *p* | ß | | 95% CI | *p* |
| (Intercept) | -0.02 | -0.17 – 0.13 | 0.758 | -0.04 | | -0.11 – 0.04 | 0.327 |
| Time | -0.14 | -0.22 – -0.05 | **0.001** | -0.21 | | -0.25 – -0.17 | **<0.001** |
| Empathic disconnection | -0.42 | -0.68 – -0.16 | **0.002** | 0.04 | | -0.09 – 0.17 | 0.521 |
| Age | -0.07 | -0.35 – 0.21 | 0.606 | -0.24 | | -0.37 – -0.10 | **<0.001** |
| Gender [male] | 0.01 | -0.30 – 0.33 | 0.929 | -0.18 | | -0.32 – -0.04 | **0.012** |
| Gender [other] | 0.72 | -0.55 – 2.00 | 0.268 | 1.44 | | 0.79 – 2.08 | **<0.001** |
| Time x empathic disconnection | 0.19 | 0.02 – 0.36 | **0.029** | 0.06 | | -0.02 – 0.14 | 0.135 |
| Random effects | | | | | | | |
| σ^2^ | 0.09 | | | | 0.07 | | |
| τ_00_ | 0.75 _id_ | | | | 0.45 _id_ | | |
| ICC | 0.90 | | | | 0.86 | | |
| N | 194 _id_ | | | | 545 _id_ | | |
| Observations | 517 | | | | 1620 | | |
| Marginal R^2^ / conditional R^2^ | 0.081 / 0.907 | | | | 0.089 / 0.874 | | |
| *Note.* ß = standardized regression estimates. | | | | | | | |

| Table S6  *Analysis of Social Isolation Model Separated Per Group* | | | | | | | |
| --- | --- | --- | --- | --- | --- | --- | --- |
|  | **With pre-existing diagnosis** | | | | **Without pre-existing diagnosis** | | |
| Predictors | ß | 95% CI | *p* | | ß | 95% CI | *p* |
| (Intercept) | -0.02 | -0.16 – 0.12 | 0.812 | | -0.07 | -0.13 – 0.00 | 0.050 |
| Time | 0.06 | -0.04 – 0.15 | 0.234 | | -0.09 | -0.13 – -0.04 | **<0.001** |
| Social isolation | 0.59 | 0.47 – 0.71 | **<0.001** | | 0.43 | 0.37 – 0.48 | **<0.001** |
| Age | -0.06 | -0.32 – 0.20 | 0.670 | | -0.19 | -0.31 – -0.08 | **0.001** |
| Gender [male] | -0.15 | -0.44 – 0.14 | 0.310 | | -0.12 | -0.24 – -0.00 | **0.044** |
| Gender [other] | 1.31 | 0.13 – 2.49 | **0.029** | | 1.29 | 0.71 – 1.86 | **<0.001** |
| Household members | -0.04 | -0.25 – 0.17 | 0.679 | | 0.04 | -0.06 – 0.14 | 0.405 |
| Digital contact | 0.03 | -0.04 – 0.11 | 0.413 | | -0.01 | -0.05 – 0.04 | 0.794 |
| Real-life contact | -0.02 | -0.12 – 0.07 | 0.611 | | -0.01 | -0.05 – 0.04 | 0.756 |
| Time x social isolation | 0.15 | -0.01 – 0.32 | 0.072 | | -0.11 | -0.19 – -0.03 | **0.006** |
| Random effects | | | | | | | |
| σ^2^ | 0.08 | | | 0.07 | | | |
| τ_00_ | 0.65 _id_ | | | 0.35 _id_ | | | |
| ICC | 0.89 | | | 0.83 | | | |
| N | 194 _id_ | | | 543 _id_ | | | |
| Observations | 517 | | | 1609 | | | |
| Marginal R^2^ / conditional R^2^ | 0.143 / 0.901 | | | 0.174 / 0.861 | | | |
| *Note.* ß = standardized regression estimates. | | | | | | | |

## Contrast Analyses

We analyzed trends and within- and between-group differences of distress levels with emmeans package (Lenth et al., 2020) using *emtrends()* and *emmeans()* functions. Tables S7, S9, and S11 display the predicted time trends of psychological distress for different levels of social factor levels (low, average, and high) and depending on group (pre-existing diagnosis vs. no pre-existing diagnosis). Here, we did not calculate *p*-values, but lower and upper confidence levels are displayed indicating significant time trends when not including 0.

Thus, we were able to do pairwise comparisons between estimated marginal means of our outcome variable (psychological distress) while taking the Social Factor x Group (pre-existing diagnosis vs. no diagnosis) interaction into account. Within- and between group comparisons of these averaged distress levels for each social factor level (low, average, and high) are displayed in Tables S8, S10, and S12. For instance, the contrast “4.84 Diagnosis − 6.7 Diagnosis” suggests that individuals with diagnosis differ significantly in their estimated distress levels over time (by 0.5), depending on their expression of social resources (low vs. high).

Social factors (social resources, empathic disconnection, and social isolation) were fixed at different levels (mean, mean +/− standard deviation). Statistical significance was set at a *p-*value less than .05. Results are Bonferroni corrected.

## Model 1: Social Resources Model

| Table S7  *Trend Analysis of Social Resources Model* | | | | | | | |
| --- | --- | --- | --- | --- | --- | --- | --- |
| **Social resources** | **Pre-existing diagnosis** | **Trend** | **SE** | **df** | **Lower confidence level** | **Upper confidence level** |  |
| 4.84 | Diagnosis | -0.000 | 0.000 | Inf | -0.001 | 0.000 |  |
| 5.77 | Diagnosis | -0.002 | 0.000 | Inf | -0.002 | -0.001 | ***** |
| 6.70 | Diagnosis | -0.003 | 0.000 | Inf | -0.004 | -0.002 | ***** |
| 4.84 | No Diagnosis | -0.002 | 0.000 | Inf | -0.003 | -0.002 | ***** |
| 5.77 | No Diagnosis | -0.002 | 0.000 | Inf | -0.002 | -0.001 | ***** |
| 6.70 | No Diagnosis | -0.001 | 0.000 | Inf | -0.001 | -0.001 | ***** |
| *Note.* Results are averaged over the levels of gender; Contrasts are Bonferroni corrected; * marks significant time trend. | | | | | | | |

| Table S8  *Social Resources Model: Pairwise Comparisons of Predicted Distress Level* | | | | | |
| --- | --- | --- | --- | --- | --- |
| **Contrast: Pre-existing diagnosis x social resources** | **Estimate** | **SE** | **df** | **z. ratio** | ***p*** |
| Within-group comparisons |  |  |  |  |  |
| 4.84 Diagnosis − 5.77 Diagnosis | 0.250 | 0.023 | Inf | 10.825 | **< 0.001** |
| 4.84 Diagnosis − 6.7 Diagnosis | 0.500 | 0.046 | Inf | 10.825 | **< 0.001** |
| 5.77 Diagnosis − 6.7 Diagnosis | 0.250 | 0.023 | Inf | 10.825 | **< 0.001** |
| 4.84 No Diagnosis − 5.77 No Diagnosis | 0.138 | 0.015 | Inf | 9.055 | **< 0.001** |
| 4.84 No Diagnosis − 6.7 No Diagnosis | 0.275 | 0.030 | Inf | 9.055 | **< 0.001** |
| 5.77 No Diagnosis − 6.7 No Diagnosis | 0.138 | 0.015 | Inf | 9.055 | **< 0.001** |
| Between-group comparisons |  |  |  |  |  |
| 4.84 Diagnosis − 4.84 No Diagnosis | 0.399 | 0.038 | Inf | 10.514 | **< 0.001** |
| 4.84 Diagnosis − 5.77 No Diagnosis | 0.536 | 0.034 | Inf | 15.843 | **< 0.001** |
| 4.84 Diagnosis − 6.7 No Diagnosis | 0.674 | 0.036 | Inf | 18.583 | **< 0.001** |
| 5.77 Diagnosis − 4.84 No Diagnosis | 0.149 | 0.033 | Inf | 4.512 | **< 0.001** |
| 5.77 Diagnosis − 5.77 No Diagnosis | 0.286 | 0.028 | Inf | 10.190 | **< 0.001** |
| 5.77 Diagnosis − 6.7 No Diagnosis | 0.424 | 0.031 | Inf | 13.730 | **< 0.001** |
| 6.7 Diagnosis – 4.84 No Diagnosis | -0.101 | 0.042 | Inf | -2.382 | 0.258 |
| 6.7 Diagnosis − 5.77 No Diagnosis | 0.036 | 0.039 | Inf | 0.940 | 1.000 |
| 6.7 Diagnosis − 6.7 No Diagnosis | 0.174 | 0.041 | Inf | 4.273 | **< 0.001** |
| *Note.* Results are averaged over the levels of gender; levels for social resources are fixed at mean = 5.77, mean + SD = 6.7, mean − SD = 4.84; *p-*values are Bonferroni corrected. | | | | | |

## Model 2: Empathic Disconnection Model

| Table S9  *Trend Analysis of Empathic Disconnection Model* | | | | | | | |
| --- | --- | --- | --- | --- | --- | --- | --- |
| **Empathic disconnection** | **Pre-existing diagnosis** | **Trend** | **SE** | **df** | **Lower confidence level** | **Upper confidence level** |  |
| 1.57 | Diagnosis | -0.003 | 0.000 | Inf | -0.003 | -0.002 | ***** |
| 2.15 | Diagnosis | -0.001 | 0.000 | Inf | -0.002 | -0.001 | ***** |
| 2.73 | Diagnosis | -0.000 | 0.000 | Inf | -0.001 | 0.001 |  |
| 1.57 | No Diagnosis | -0.002 | 0.000 | Inf | -0.002 | -0.001 | ***** |
| 2.15 | No Diagnosis | -0.002 | 0.000 | Inf | -0.002 | -0.001 | ***** |
| 2.73 | No Diagnosis | -0.001 | 0.000 | Inf | -0.002 | -0.001 | ***** |
| *Note.* Results are averaged over the levels of gender; Contrasts are Bonferroni corrected; * marks significant time trend. | | | | | | | |

| Table S10  *Empathic Disconnection Model: Pairwise Comparisons of Predicted Distress Level* | | | | | |
| --- | --- | --- | --- | --- | --- |
| **Contrast: Pre-existing diagnosis x empathic disconnection** | **Estimate** | **SE** | **df** | **z. ratio** | ***p*** |
| Within-group comparisons |  |  |  |  |  |
| 1.57 Diagnosis − 2.15 Diagnosis | 0.130 | 0.027 | Inf | 4.863 | **< 0.001** |
| 1.57 Diagnosis − 2.73 Diagnosis | 0.259 | 0.053 | Inf | 4.863 | **< 0.001** |
| 2.15 Diagnosis − 2.73 Diagnosis | 0.130 | 0.027 | Inf | 4.863 | **< 0.001** |
| 1.57 No Diagnosis − 2.15 No Diagnosis | -0.006 | 0.016 | Inf | -0.383 | 1.000 |
| 1.57 No Diagnosis − 2.73 No Diagnosis | -0.012 | 0.032 | Inf | -0.383 | 1.000 |
| 2.15 No Diagnosis − 2.73 No Diagnosis | -0.006 | 0.016 | Inf | -0.383 | 1.000 |
| Between-group comparisons |  |  |  |  |  |
| 1.57 Diagnosis − 1.57 No Diagnosis | 0.456 | 0.042 | Inf | 10.986 | **< 0.001** |
| 1.57 Diagnosis − 2.15 No Diagnosis | 0.450 | 0.038 | Inf | 11.815 | **< 0.001** |
| 1.57 Diagnosis − 2.73 No Diagnosis | 0.444 | 0.041 | Inf | 10.798 | **< 0.001** |
| 2.15 Diagnosis − 1.57 No Diagnosis | 0.327 | 0.034 | Inf | 9.492 | **< 0.001** |
| 2.15 Diagnosis − 2.15 No Diagnosis | 0.321 | 0.030 | Inf | 10.753 | **< 0.001** |
| 2.15 Diagnosis − 2.73 No Diagnosis | 0.314 | 0.033 | Inf | 9.456 | **< 0.001** |
| 2.73 Diagnosis − 1.57 No Diagnosis | 0.197 | 0.045 | Inf | 4.334 | **< 0.001** |
| 2.73 Diagnosis − 2.15 No Diagnosis | 0.191 | 0.042 | Inf | 4.565 | **< 0.001** |
| 2.73 Diagnosis − 2.73 No Diagnosis | 0.185 | 0.044 | Inf | 4.192 | **< 0.001** |
| *Note.* Results are averaged over the levels of gender; levels for empathic disconnection are fixed at mean = 2.15, mean + SD = 2.73, mean − SD = 1.57; *p-*values are Bonferroni corrected. | | | | | |

## Model 3: Social Isolation Model

| Table S11  *Trend Analysis of Social Isolation Model* | | | | | | | |
| --- | --- | --- | --- | --- | --- | --- | --- |
| **Social isolation** | **Pre-existing diagnosis** | **Trend** | **SE** | **df** | **Lower confidence level** | **Upper confidence level** |  |
| 0.53 | Diagnosis | -0.000 | 0.000 | Inf | -0.001 | 0.000 |  |
| 1.52 | Diagnosis | 0.000 | 0.000 | Inf | -0.000 | 0.001 |  |
| 2.51 | Diagnosis | 0.001 | 0.000 | Inf | 0.000 | 0.002 | ***** |
| 0.53 | No Diagnosis | -0.000 | 0.000 | Inf | -0.001 | 0.000 |  |
| 1.52 | No Diagnosis | -0.001 | 0.000 | Inf | -0.001 | -0.000 | ***** |
| 2.51 | No Diagnosis | -0.001 | 0.000 | Inf | -0.002 | -0.001 | ***** |
| *Note.* Results are averaged over the levels of gender; Contrasts are Bonferroni corrected; * marks significant time trend. | | | | | | | |

| Table S12  *Social Isolation Model: Pairwise Comparisons of Predicted Distress Level* | | | | | |
| --- | --- | --- | --- | --- | --- |
| **Contrast: Pre-existing diagnosis x social isolation** | **Estimate** | **SE** | **df** | **z. ratio** | ***p*** |
| Within-group comparisons |  |  |  |  |  |
| 0.53 Diagnosis − 1.52 Diagnosis | -0.174 | 0.012 | Inf | -14.584 | **< 0.001** |
| 0.53 Diagnosis − 2.51 Diagnosis | -0.348 | 0.024 | Inf | -14.584 | **< 0.001** |
| 1.52 Diagnosis − 2.51 Diagnosis | -0.174 | 0.012 | Inf | -14.584 | **< 0.001** |
| 0.53 No Diagnosis − 1.52 No Diagnosis | -0.084 | 0.007 | Inf | -12.396 | **< 0.001** |
| 0.53 No Diagnosis − 2.51 No Diagnosis | -0.168 | 0.014 | Inf | -12.396 | **< 0.001** |
| 1.52 No Diagnosis − 2.51 No Diagnosis | -0.084 | 0.007 | Inf | -12.396 | **< 0.001** |
| Between-group comparisons |  |  |  |  |  |
| 0.53 Diagnosis − 0.53 No Diagnosis | 0.179 | 0.032 | Inf | 5.527 | **< 0.001** |
| 0.53 Diagnosis − 1.52 No Diagnosis | 0.095 | 0.032 | Inf | 2.992 | **0.042** |
| 0.53 Diagnosis − 2.51 No Diagnosis | 0.011 | 0.032 | Inf | 0.333 | 1.000 |
| 1.52 Diagnosis − 0.53 No Diagnosis | 0.352 | 0.028 | Inf | 12.486 | **< 0.001** |
| 1.52 Diagnosis − 1.52 No Diagnosis | 0.269 | 0.027 | Inf | 9.783 | **< 0.001** |
| 1.52 Diagnosis − 2.51 No Diagnosis | 0.185 | 0.028 | Inf | 6.522 | **< 0.001** |
| 2.51 Diagnosis − 0.53 No Diagnosis | 0.526 | 0.029 | Inf | 18.224 | **< 0.001** |
| 2.51 Diagnosis − 1.52 No Diagnosis | 0.442 | 0.028 | Inf | 15.743 | **< 0.001** |
| 2.51 Diagnosis − 2.51 No Diagnosis | 0.359 | 0.029 | Inf | 12.392 | **< 0.001** |
| *Note.* Results are averaged over the levels of gender; levels for social isolation are fixed at mean = 1.52, mean + SD = 2.51, mean − SD = 0.53; *p-*values are Bonferroni corrected. | | | | | |

# References

Bartels, C., Hessmann, P., Schmidt, U., Vogelgsang, J., Ruhleder, M., Kratzenberg, A., Treptow, M. et al. (2021). Medium-term and peri-lockdown course of psychosocial burden during the ongoing COVID-19 pandemic: A longitudinal study on patients with pre-existing mental disorders. *European Archives of Psychiatry and Clinical Neuroscience.* <https://doi.org/10.1007/s00406-021-01351-y>

Bendau, A., Kunas, S. L., Wyka, S., Petzold, M. B., Plag, J., Asselmann, E., and Ströhle, A. (2021). Longitudinal changes of anxiety and depressive symptoms during the COVID-19 pandemic in Germany: The role of pre-existing anxiety, depressive, and other mental disorders. *Journal of Anxiety Disorders, 79*, 102377. <https://doi.org/10.1016/j.janxdis.2021.102377>

Hofer, A., Kachel, T., Plattner, B., Chernova, A., Conca, A., Fronthaler, M., Haring, C., Holzner, B., Huber, M., Marksteiner, J., Miller, C., Pardeller, S., Perwanger, V., Pycha, R., Schmidt, M., Sperner-Unterweger, B., Tutzer, F., & Frajo-Apor, B. (2022). Mental health in individuals with severe mental disorders during the covid-19 pandemic: A longitudinal investigation. *Schizophrenia, 8*(1), 17. <https://doi.org/10.1038/s41537-022-00225-z>

Lenth, R., Buerkner, P., Herve, M., Love, J., Riebl, H., and Singmann, H. (2020). emmeans: Estimated Marginal Means, aka Least-Squares Means [R package version 1.5.1.]. *CRAN.* <https://cran.r-project.org/web/packages/emmeans>

Mergel, E., and Schützwohl, M. (2021). A longitudinal study on the COVID-19 pandemic and its divergent effects on social participation and mental health across different study groups with and without mental disorders. *Social Psychiatry and Psychiatric Epidemiology, 56*, 1459–1468. <https://doi.org/10.1007/s00127-021-02025-9>

Pan, K.-Y., Kok, A. A. L., Eikelenboom, M., Horsfall, M., Jörg, F., Luteijn, R. A., Rhebergen, D., van Oppen, P., Giltay, E. J., and Penninx, B. W. J. H. (2021). The mental health impact of the COVID-19 pandemic on people with and without depressive, anxiety, or obsessive-compulsive disorders: A longitudinal study of three Dutch case-control cohorts. *The Lancet Psychiatry, 8*, 121–129. <https://doi.org/10.1016/S2215-0366(20)30491-0>

Pierce, M., McManus, S., Hope, H., Hotopf, M., Ford, T., Hatch, S. L., John, A. et al. (2021). Mental health responses to the COVID-19 pandemic: A latent class trajectory analysis using longitudinal UK data. *The Lancet Psychiatry, 8*, 610–619. <https://doi.org/10.1016/S2215-0366(21)00151-6>

Racine, N., Hetherington, E., McArthur, B. A., McDonald, S., Edwards, S., Tough, S., and Madigan, S. (2021). Maternal depressive and anxiety symptoms before and during the COVID-19 pandemic in Canada: A longitudinal analysis. *The Lancet Psychiatry,* 8, 405–415. <https://doi.org/10.1016/S2215-0366(21)00074-2>

Robinson, E., Sutin, A. R., Daly, M., and Jones, A. (2022). A systematic review and meta-analysis of longitudinal cohort studies comparing mental health before versus during the COVID-19 pandemic in 2020. *Journal of Affective Disorders, 296*, 567–576. <https://doi.org/10.1016/j.jad.2021.09.098>

Yocum, A. K., Zhai, Y., McInnis, M. G., and Han, P. (2021). Covid-19 pandemic and lockdown impacts: A description in a longitudinal study of bipolar disorder. *Journal of Affective Disorders,* *282*, 1226–1233. <https://doi.org/10.1016/j.jad.2021.01.028>
